# Polymorphisms in *BACE2* may affect the age of onset Alzheimer's dementia in Down syndrome^☆^

**DOI:** 10.1016/j.neurobiolaging.2013.12.022

**Published:** 2014-06

**Authors:** Kin Y. Mok, Emma L. Jones, Marisa Hanney, Denise Harold, Rebecca Sims, Julie Williams, Clive Ballard, John Hardy

**Affiliations:** a Department of Molecular Neuroscience, UCL Institute of Neurology, Queen Square, London, UK; b Reta Lila Weston Institute, UCL Institute of Neurology, London, UK; c Wolfson Centre for Age-Related Diseases, King's College London, London, UK; d Northgate Hospital, Morpeth, Northumberland, UK; e MRC Centre for Neuropsychiatric Genetics and Genomics, Cardiff University, Henry Wellcome Building for Biomedical Research, Heath Park, Cardiff, UK; f The London Down Syndrome (LonDownS) Consortium, UK

**Keywords:** Down syndrome, *BACE2*, Alzheimer's disease, rs2252576

## Abstract

It is known that Alzheimer's disease (AD) presents at an early age in people with Down syndrome (DS). The trisomy 21 in DS provides an opportunity to study the effect of duplicated genes in AD. *APP* and *BACE2* are 2 genes located in chromosome 21 and related to AD. We looked into our cohort of 67 DS cases with dementia for the effect of *BACE2* variants in age of onset of dementia. Of the 83 single-nucleotide polymorphisms (SNPs), 6 were associated with age of onset and another 8 SNPs were borderline associated. Our finding also replicated a previous study showing association of rs2252576 with AD.

## 1 Introduction

Down syndrome (DS), or trisomy 21, is caused by an extra copy of chromosome 21. The prevalence of Alzheimer's disease (AD) and dementia in the general population and in people with DS increases with age (Margallo-Lana et al., 2007; Murray et al., 2013). In DS, the densities of senile plaques and neurofibrillary tangles increase with aging but can occur in people as young as 37 years (Mann et al., 1990). Despite the pathologic changes, the age of onset (AOO) of dementia varies (Prasher and Krishnan, 1993), suggesting that factors other than trisomy of *APP* in DS are important.

The study of genetic roles in AD contributes to understanding dementia in DS. We have previously shown that in DS polymorphisms in *PICALM* and *APOE* are associated with AOO of dementia, and there is a nonsignificant trend in risk allele loading derived from AD meta-analysis (Jones et al., 2013). Conversely, study of dementia in DS, particularly the role of trisomy in chromosome 21, allows us to understand mechanisms of AD. *APP* and *BACE2* are both located on chromosome 21. We previously showed that haplotypes in *BACE2* are associated with AD (Myllykangas et al., 2005). However, other studies suggested that *BACE2* might not play a significant role in AD (Cheon et al., 2008; Holler et al., 2012). We attempted to look into the role of *BACE2* in AOO of dementia in DS.

## 2 Methods

The cohort, genotyping and quality control measures were as previously reported (Jones et al., 2013). In brief, this comprised of 94 samples from 2 clinical trials (DOWNSLIT [http://public.ukcrn.org.uk/search/StudyDetail.aspx?StudyID=5927] and MEADOWS; Hanney et al., 2012) and 64 brain samples from various brain banks (details in Supplementary Data). For samples from clinical trials, AOO of dementia was as recorded by the trial psychiatrist. Mean duration of dementia before death in people with DS is approximately 5 years, and AOO for the autopsy cohort was defined as the age of death minus 5, which assumes all individuals over the age of 34 years had dementia before death.

DNA was obtained from blood or brain using the commercially available DNeasy Blood and Tissue kit (Qiagen, UK). Genotyping was done in the UCL Genomics Centre using HumanOmniExpress-12v1_H beadchips. After the initial quality control check, 129 samples remained (males: 72; females: 57). Of these, only 70 samples were classified as having dementia and only 67 samples were AOO documented. This formed the cohort for current analysis and was as previously reported (Jones et al., 2013) (details in Supplementary Table 1).

Clustering of genotypes for autosomal chromosomes in GenomeStudio (Illumina, San Diego, CA, USA) is based on diploid status. Manual recluster for polyploidy-genotyping was performed using GenomeStudio module v1.9.4 (http://res.illumina.com/documents/products/technotes/technote_genomestudio_polyploid_genotyping.pdf). All single-nucleotide polymorphisms (SNPs) (n = 85) within the *BACE2* locus +/− 50 kb were reclustered and the genotypes of SNPs were exported for further analysis. The minor allele was evaluated to be the risk allele.

Regression analysis was performed in R v2.15.2 (R Development Core Team, 2008). AOO regression was analyzed using an additive model. Components 1 and 2 in PLINK, multidimensional scaling (Purcell et al., 2007) were used as covariates, in addition to gender and the classification of the sample as clinical or autopsy. A nominal *p* value of 0.05 was used as a cutoff for nominal significance.

## 3 Results

The 67 cases (men:women = 36:31) had average age of 55.6 years (standard deviation ± 7.3). There was a total of 85 SNPs within 50 kb of *BACE2* (chr21:42539728–42654461, hg19). Of these, 2 SNPs were excluded because they were monomorphic (Supplementary Table 1).

**Fig. 1:**
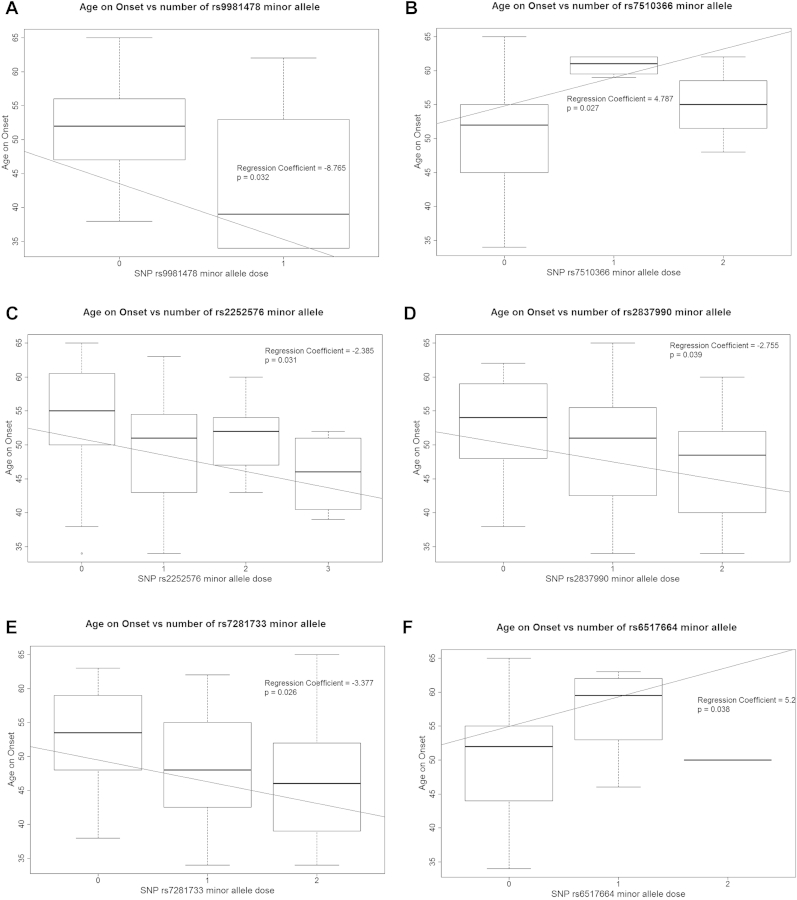
Plot of 6 SNPs with significant association with AOO around *BACE2*. Median age of onset of dementia according to genotype. 0 = homozygous major allele, 1 = 2 major alleles and 1 minor allele, 2 = 1 major allele and 2 minor alleles, and 3 = homozygous trisomy of minor allele. (A) rs9981478 C/T (minor/major); (B) rs7510366 T/C; (C) rs2252576 T/C; (D) rs2837990 A/G; (E) rs7281733 A/G; and (F) rs6517664 T/C. Abbreviations: AOO, age of onset; SNP, single-nucleotide polymorphism.

Among the 83 SNPs, 6 SNPs have a nominal significance with *p* < 0.05 and another 8 SNPs have *p* value between 0.05 and 0.1 (Fig. 1, Table 1, and Supplementary Table 1). All except 4 SNPs were clustered within the exons 6–9 and the 3′ region (Supplementary Fig. 1), which fell within a haplotype block of Hapmap3 European cohort (Supplementary Fig. 2). The most significant SNP, rs7287133 is in linkage disequilibrium (LD) with some other significant SNPs and borderline significant SNPs (Supplementary Fig. 1), suggesting that the haplotype in this region is probably important for AD.

## 4 Discussion

There are 2 major limitations in the present study. The first one is the small sample size and therefore limits the power to conclude definitely the association. The second one comes from correction for multiple testing. Multiple testing is performed for all SNPs within *BACE2*.

Correction for multiple testing within high LD block can be difficult. Applying Bonferroni correction is too conservative and leads to false negative results. HapMap Utah residents (Centre d′Etude du Polymorphisme Humain) with Northern and Western European ancestry and Toscani in Italy (CEU + TSI) data suggest the *BACE2* region falls into 5 major blocks (Supplementary Fig. 2). Most of the nominal and borderline significant SNPs cluster around the exon 6 to the 3′ untranslated region (3′UTR), and Hapmap data suggest that this is a block of high LD. If we apply the correction according to the number of haplotype blocks, the corrected *p* value of significance should be 0.05/5 = 0.01. Our SNPs will fall out of significance but are still borderline significant.

Another theoretical correction has to be made for testing multiple genes. Our previous report suggested *PICALM* and *APOE* as risk factors for early onset of dementia in DS (Jones et al., 2013). In the previous study, we tested 157 SNPs in 74 gene regions (35 SNPs in 15 genes are among the *APOE* block). The present study is focused on *BACE2* with the genotype manual reclustered for trisomy as mentioned in section 2, and can be viewed as a complimentary study to previous study. Straight multiple testing correction will result in the currently studied SNPs becoming insignificant.

The current data on DS support our group's previous report (Myllykangas et al., 2005) of association of haplotypes in *BACE2* with AD. In a study by Myllykangas, we found rs2252576 (minor T allele) and haplotypes including this SNP were associated with AD in Finns and Americans. Our current AOO regression data also show that rs2252576-T allele possibly associates with an earlier age of onset of dementia (slope = −2.4 year/per T allele) in DS.

The role of *BACE2* in AD has been controversial. *BACE2* expression was not found to be increased in adult DS brain (Cheon et al., 2008). However, Lockstone et al. (2007) found increased *BACE2* expression in adult DS brain. Holler et al. (2012) reported an increase in *BACE2* messenger RNA in DS that did not translate into increased *BACE2* protein levels and activities in DS, despite increases in other neurodegenerative diseases. However, overexpression of *BACE2* was found to decrease amyloid beta production in neuronal cultures (Abdul-Hay et al., 2012; Sun et al., 2006).

Our SNPs with nominal and borderline significance were observed mainly around the 3′ end and not in the 5′UTR region. This is in congruent with a negative association of SNPs in 5′-flanking region done in an Asian population of AD (Yu and Jia, 2009).

Association of SNPs in *BACE2* with AD is not found in the recent mega meta-analyses of genome-wide association study (Hollingworth et al., 2011; Lambert et al., 2013; Naj et al., 2011). The discrepancy may be related to our study population being all trisomy 21, including *APP*.

We postulate that with duplication of *APP*, the increased gene dosage contributes a greater role in the pathogenesis of AD, compared with other AD cases without *APP* duplication. Subsequently, gene products, for example from *BACE2*, that interact with the *APP* will play a greater role in dementia pathogenesis among trisomy cases than in AD subjects with diploid *APP*. Expression of *BACE2* was reported to be increased in adult brain with DS (Lockstone et al., 2007). The upregulation of the *APP*-related system in DS may explain why *BACE2* SNPs are not found to be associated in general AD genome-wide association study, but are suggestive when the dementia cohort is made up of *APP* trisomy, as in the present study.

Our data on rs2252576, rs2837990, and rs7281733 suggest that the minor alleles may predispose to early onset of dementia, implying these variants and the functional alteration caused are pathogenic. The opposite was found with rs7510366 and rs6517664. Pathogenicity can be because of variants enhancing a pathogenic role of the protein or diminishing a protective role. Hence, other studies are needed to determine the functional role of *BACE2*.

In summary, we find variants in *BACE2* that possibly affect the AOO of dementia in DS, suggesting a potential role of *BACE2* in AD and DS. Dementia in DS can provide an important model to study the role of *APP* and related genes in AD.

## Disclosure statement

Julie Williams has research collaborations with Elly-Lilly and Population Genetics Technologies Ltd (Cambridge) and has presented papers at conferences funded by Esai. The other authors have no conflicting financial, personal, or professional interests.

## Figures and Tables

**Table 1:** SNPs with significant association with AOO around *BACE2*

	SNP	Position	Minor allele	Nominal *p* value
Nominal significant	rs9981478	42558694	C	0.0316
	rs7510366	42581927	T	0.0274
	rs2252576	42615293	T	0.0315
	rs2837990	42620149	A	0.0394
	rs7281733	42655515	A	0.0258
	rs6517664	42704983	T	0.0376
Borderline significant	rs6517656	42583738	A	0.0866
	rs2837994	42624124	A	0.091
	rs11702001	42627969	A	0.0722
	rs8133778	42642038	A	0.075
	rs2838003	42649357	C	0.0774
	rs2006737	42657187	G	0.0793
	rs1072869	42657548	C	0.0607
	rs9984207	42662259	T	0.0601

Key: AOO, age of onset; SNP, single-nucleotide polymorphism.
