# Erratum to “Polymorphisms in *BACE2* may affect the age of onset Alzheimer's dementia in Down syndrome” [Neurobiol. Aging 35 (2014) 1513.e1–1513.e5]

**DOI:** 10.1016/j.neurobiolaging.2014.06.017

**Published:** 2014-11

**Authors:** Kin Y. Mok, Emma L. Jones, Marisa Hanney, Denise Harold, Rebecca Sims, Julie Williams, Clive Ballard, John Hardy

**Affiliations:** a Department of Molecular Neuroscience, UCL Institute of Neurology, Queen Square, London, UK; b Reta Lila Weston Institute, UCL Institute of Neurology, London, UK; c Wolfson Centre for Age-Related Diseases, King’s College London, London, UK; d Northgate Hospital, Morpeth, Northumberland, UK; e MRC Centre for Neuropsychiatric Genetics and Genomics, Cardiff University, Henry Wellcome Building for Biomedical Research, Heath Park, Cardiff, UK; f The London Down Syndrome (LonDownS) Consortium, UK

The following statement should appear in the Acknowledgements on page 1513.e4:

“CB and EJ would like to acknowledge the support of the National Institute for Health Research (NIHR) Mental Health Biomedical Research Centre and Dementia Unit at South London and Maudsley NHS Foundation Trust and [Institute of Psychiatry] King’s College London. This article presents independent research partially-supported by the National Institute for Health Research (NIHR). The views expressed are those of the author(s) and not necessarily those of the NHS, the NIHR or the Department of Health.”

