# Erratum to “Polymorphisms in *BACE2* may affect the age of onset Alzheimer's dementia in Down syndrome” [Neurobiol. Aging 35 (2014) 1513.e1–1513.e5]

**DOI:** 10.1016/j.neurobiolaging.2014.08.026

**Published:** 2014-12

**Authors:** Kin Y. Mok, Emma L. Jones, Marisa Hanney, Denise Harold, Rebecca Sims, Julie Williams, Clive Ballard, John Hardy

In the above-mentioned article, an error in the text on page 1513.e3 has been noted. In the second paragraph of the section “RESULTS”, the last sentence says,

“The most significant SNP, rs7287133 is in linkage disequilibrium (LD) with some other significant SNPs and borderline significant SNPs (Supplementary Fig. 1), suggesting that the haplotype in this region is probably important for AD”.

This is incorrect, and the corrected text should read

“The most significant SNP, rs7281733 is in linkage disequilibrium (LD) with some other significant SNPs and borderline significant SNPs (Supplementary Fig. 1), suggesting that the haplotype in this region is probably important for AD”.

