# Supplementary material for: Polymorphisms in BACE2 may affect the age of onset Alzheimer's dementia in Down syndrome
Source: Neurobiol Aging. 2014 Jun;35(6):1513.e1–5. doi: 10.1016/j.neurobiolaging.2013.12.022 (PMC3969241; doi:10.1016/j.neurobiolaging.2013.12.022)
Supplement: Supplementary Materials [file mmc1.pdf]

Supplement 1 Details of brain bank sample

The Newcastle and London Neurodegenerative Disease brain banks,

Thomas Willis Oxford Brain Collection, as part of the Brains for Dementia Research initiative,

the Medical Research Council Alzheimer Brain & Tissue Bank in Edinburgh,

the NICHD Brain and Tissue Bank for Developmental Disorders, University of Maryland (NICHD Contract No. N01-HD-4-3368 and NO1-HD-4-3383),

the Alzheimer's Disease Research Center, Washington University in St. Louis,

and the Netherlands Brain Bank.

Supplement 2 (Method

The data were assembled in Genome Studio (Illumina, San Diego, CA). A total of 158 samples were genotyped. After the initial quality control check, 129 samples (72 male and 57 female) and 642,251 SNPs remained.

SNPs were first curated in Genome Studio. Those SNPs in Chr 1 to 22 that failed the call frequency parameter (defined as <0.98) were analyzed by re-cluster in Genome Studio. Within this group, only those SNPS that passed quality parameters were kept (namely Het excess between 0.1 and −0.1 and cluster separation >0.3). All others were discarded (zeroed in Genome Studio). Samples with call rates <0.975 after this process were excluded (9 samples and 29,537 SNPs were excluded in this step).

Samples were curated in PLINK (v1.07) (Purcell, et al., 2007) for gender check and relatedness (defined as PiHat >0.125), and SNPs were checked for minor allele frequency (≥0.01), haplotype missingness (p < 10e-4), and Hardy–Weinburg Equilibrium (p < 10e-6). Ten samples were excluded (6 were duplicates of another sample in the study). 58,737 SNPs were excluded at this stage, of which 58,405 were due to MAF <0.01. The outcome of this process resulted in 139 samples (61 female and 78 male) and 642,251 SNPs for further analysis.

Samples were merged with Hapmap data. MultiDimensional Scaling on the combined samples was used for assessing population outliers. Those samples that were 4 standard deviations away from the mean of CEU/TSI combined data were excluded. Ten samples were found to be nonwhite or mixed. At this stage, only 129 samples (72 male and 57 female) and 642,251 SNPs remained.

As dementia develops at a mean age of 50 to 55 years, and in our clinical sample series, the earliest age of dementia onset was 34 years, we excluded all cases with age <34 at their last cognitive assessment or autopsy. This resulted in 120 valid samples with 70 having dementia (clinically defined, or belonging to the autopsy cohort). Among the 70 dementia cases, 3 had no AAO in the record. The

analysis was based on 67 cases (Table 1). The youngest age in the autopsy series was 39, and therefore AAO in this case was 34 as defined above. There were no differences in gender frequencies between the clinical and autopsy samples used at this stage ($\chi2 = 0.005$, $p = 0.946$) or age (Mann–Whitney U test, $p = 0.419$).

We then went back to the raw data within GenomeStudio and manually re-clustered the 85 SNPs within 50kb of *BACE2,* as per recommendation in the Illumina polypoid genotyping [http://res.illumina.com/documents/products/technotes/technote_genomestudio_polyploid_genotyping.pdf]. Genotypes were re-clustered into AAA, AAB, ABB, BBB. Data of the 85 SNPs from the 67 samples with AOO were exported for analysis. Of these, 2 SNPs were monomorphic and not entered into analysis.

Regression analyses were based on the minor allele frequency as risk allele.
