# Supplementary material for: Polymorphisms in BACE2 may affect the age of onset Alzheimer's dementia in Down syndrome
Source: Neurobiol Aging. 2014 Jun;35(6):1513.e1–5. doi: 10.1016/j.neurobiolaging.2013.12.022 (PMC3969241; doi:10.1016/j.neurobiolaging.2013.12.022)
Supplement: Supplementary Table 1 [file mmc2.docx]

**Supplementary table 1**

All SNPs (HumanOmniExpress-12v1_H) within 50kb of *BACE2* and P value of regression

| SNP | Position (hg19) | minor allele | P value |  |
| --- | --- | --- | --- | --- |
| rs13050861 | 42485808 | A | 0.586 |  |
| rs2837958 | 42492113 | A | 0.842 |  |
| rs9979680 | 42497458 | G | 0.357 |  |
| rs4818209 | 42501062 | G | 0.734 |  |
| rs13049109 | 42502830 | T | 0.621 |  |
| rs1571729 | 42504113 | A | 0.839 |  |
| rs965494 | 42509823 | T | 0.321 |  |
| rs10470196 | 42510744 | A | 0.83 |  |
| rs2837960 | 42511918 | G | 0.752 |  |
| rs1001453 | 42515277 | T | 0.798 |  |
| rs2007397 | 42516192 | A | 0.328 |  |
| rs9984070 | 42516327 | T | 0.492 |  |
| rs1571733 | 42518034 | A | 0.585 |  |
| rs2837961 | 42518599 | C | 0.647 |  |
| rs2837964 | 42520646 | A | 0.196 |  |
| rs9974272 | 42521711 | G | 0.282 |  |
| rs11701851 | 42523852 | T | 0.403 |  |
| rs11702752 | 42524741 | C | 0.398 |  |
| rs10154234 | 42525007 | A | 0.403 |  |
| rs2016240 | 42530260 | T | 0.426 |  |
| rs7276900 | 42532075 | A | 0.854 |  |
| rs9981347 | 42543537 | T | 0.711 |  |
| rs734757 | 42543663 | T | 0.51 |  |
| rs1467756 | 42545250 | C | 0.929 |  |
| rs766850 | 42546817 | A | 0.931 |  |
| rs4818219 | 42547894 | G | 0.867 |  |
| rs9977003 | 42548721 | A | 0.831 |  |
| rs11909439 | 42554042 | T | 0.288 |  |
| rs7278659 | 42555005 | A | 0.689 |  |
| rs9981478 | 42558694 | C | 0.0316 | * |
| rs8133241 | 42561034 | G | 0.597 |  |
| rs914185 | 42567137 | T | 0.456 |  |
| rs4816713 | 42574184 | T | 0.349 |  |
| rs2837967 | 42576964 | A | 0.331 |  |
| rs746064 | 42581448 | A | 0.433 |  |
| rs2410406 | 42581529 | C | 0.616 |  |
| rs737287 | 42581703 | A | 0.628 |  |
| rs7510366 | 42581927 | T | 0.0274 | * |
| rs6517656 | 42583738 | A | 0.0866 | # |
| rs13052926 | 42585088 | G | 0.203 |  |
| rs9983496 | 42586130 | C | 0.682 |  |
| rs2837968 | 42586604 | T | 0.939 |  |
| rs7280770 | 42595890 | A | 0.659 |  |
| rs7278856 | 42598887 | G | 0.734 |  |
| rs2837974 | 42602735 | G | 0.236 |  |
| rs2009135 | 42608231 | C | 0.552 |  |
| rs2252576 | 42615293 | T | 0.0315 | * |
| rs2837981 | 42616005 | C | 0.513 |  |
| rs3827211 | 42619254 | G | 0.929 |  |
| rs2837988 | 42619544 | A | 0.635 |  |
| rs914186 | 42619657 | G | 0.367 |  |
| rs914187 | 42619749 | T | 0.655 |  |
| rs2837990 | 42620149 | A | 0.0394 | * |
| rs2837992 | 42620520 | T | 0.244 |  |
| rs1046210 | 42622786 | T | 0.732 |  |
| rs2837994 | 42624124 | A | 0.091 | # |
| rs2837996 | 42626706 | T | 0.551 |  |
| rs11702001 | 42627969 | A | 0.0722 | # |
| rs2837998 | 42630093 | G | 0.123 |  |
| rs7277420 | 42637215 | T | 0.813 |  |
| rs8133778 | 42642038 | A | 0.075 | # |
| rs2838000 | 42646518 | A | 0.666 |  |
| rs12149 | 42647821 | T | 0.448 |  |
| rs2838003 | 42649357 | C | 0.0774 | # |
| rs3746889 | 42651946 | C | 0.141 |  |
| rs8126886 | 42652717 | T | 0.303 |  |
| rs1964926 | 42653121 | A | 0.317 |  |
| rs1999331 | 42654497 | A | 0.211 |  |
| rs7281733 | 42655515 | A | 0.0258 | * |
| rs2006737 | 42657187 | G | 0.0793 | # |
| rs1072869 | 42657548 | C | 0.0607 | # |
| rs2410416 | 42658473 | G | 0.225 |  |
| rs4818230 | 42659676 | G | 0.61 |  |
| rs9984207 | 42662259 | T | 0.0601 | # |
| rs6517662 | 42674022 | A | 0.221 |  |
| rs10439675 | 42681597 | C | 0.779 |  |
| rs2838007 | 42682644 | C | 0.847 |  |
| rs12626413 | 42683660 | G | 0.847 |  |
| rs2838008 | 42685044 | T | 0.675 |  |
| rs7283900 | 42689700 | G | 0.57 |  |
| rs2838012 | 42694870 | A | 0.495 |  |
| rs441810 | 42698907 | G | 0.778 |  |
| rs6517664 | 42704983 | T | 0.0376 | * |
| * nominal p<0.05,  # nominal p< 0.1 | |  |  |  |
| Monomorphic SNPs: rs10211865 and rs2837980 | | | | |
|  |  |  |  |  |
|  |  |  |  |  |
