# Supplementary material for: Polymorphisms in BACE2 may affect the age of onset Alzheimer's dementia in Down syndrome
Source: Neurobiol Aging. 2014 Jun;35(6):1513.e1–5. doi: 10.1016/j.neurobiolaging.2013.12.022 (PMC3969241; doi:10.1016/j.neurobiolaging.2013.12.022)
Supplement: Supplementary Figure 1 [file mmc3.pdf]

Supplementary Figure 1

Plot of regression p value in AOO of dementia in Down syndrome in the 83 SNPs around *BACE2*

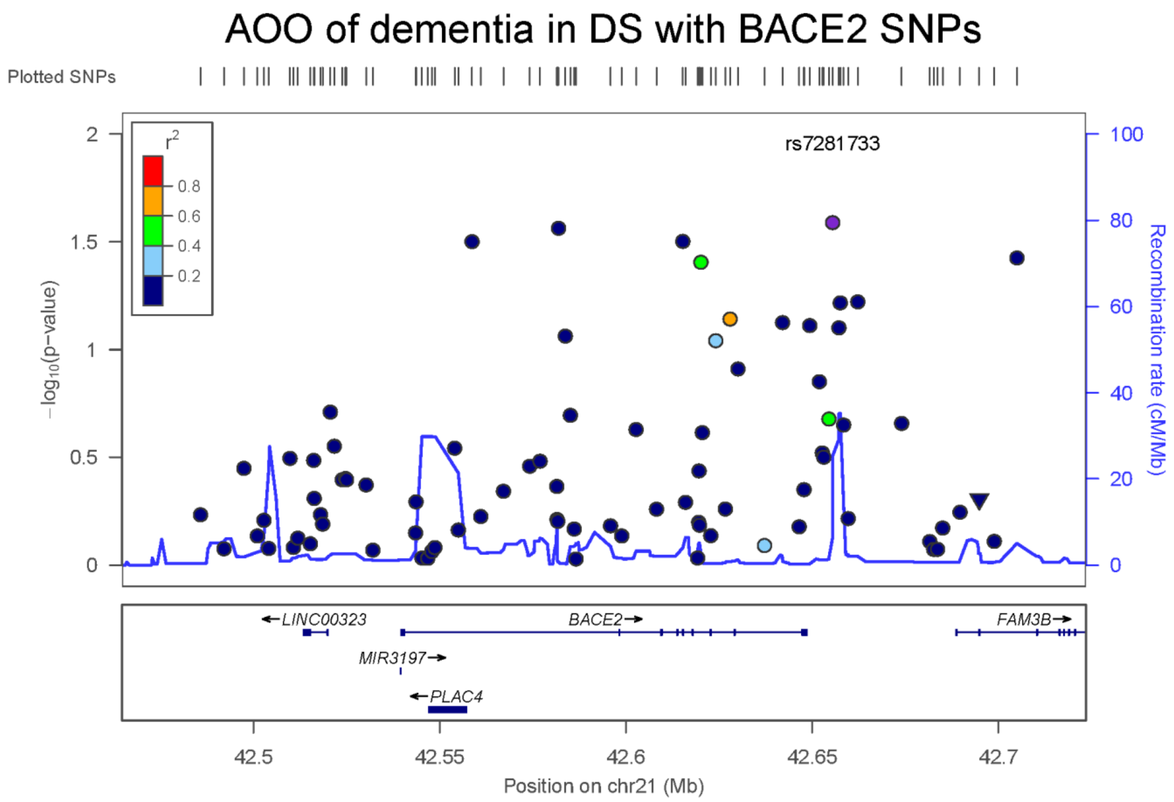

More nominally or borderline significant SNPs are found around the exon 7-9 and immediate 3'UTR region.
