# Supplementary material for: Polymorphisms in BACE2 may affect the age of onset Alzheimer's dementia in Down syndrome
Source: Neurobiol Aging. 2014 Jun;35(6):1513.e1–5. doi: 10.1016/j.neurobiolaging.2013.12.022 (PMC3969241; doi:10.1016/j.neurobiolaging.2013.12.022)
Supplement: Supplementary Figure 2 [file mmc4.pdf]

Supplementary Figure 2

Plot of LD around *BACE2* region using Haploview 4.2 using data from HapMap3 European population (Barrett, et al., 2005)

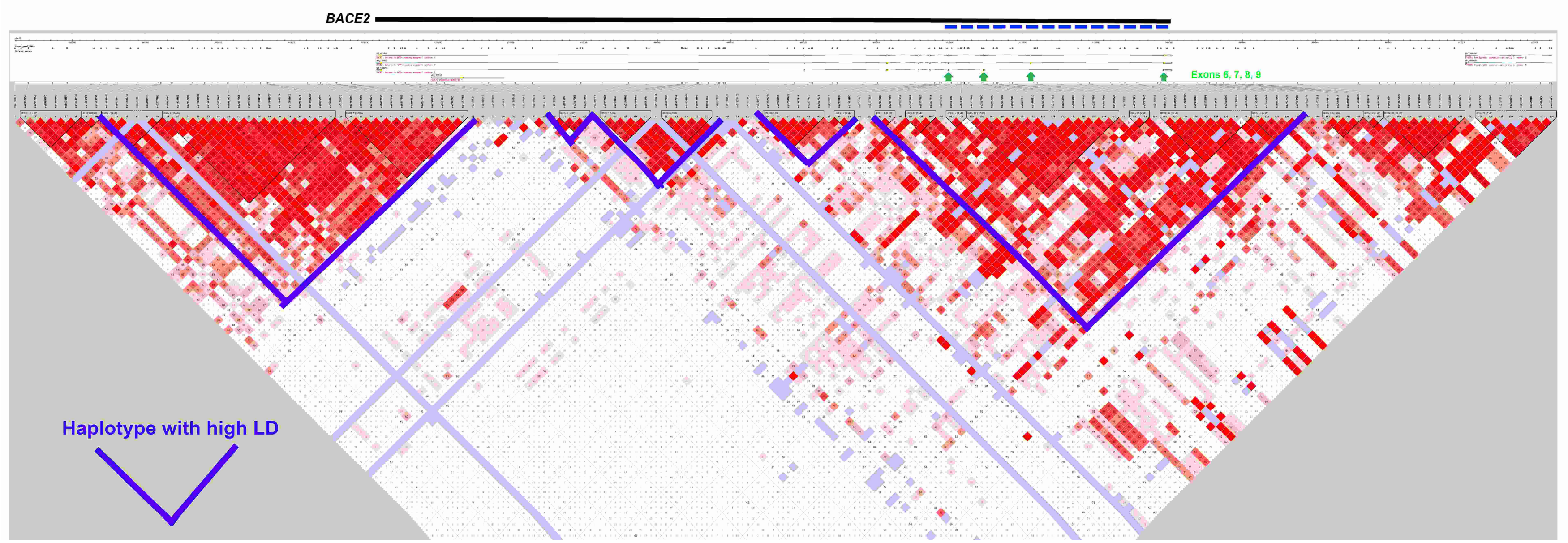

The blue triangles show blocks with high LD. Exon 6-9 fall into a block

Barrett, J.C., Fry, B., Maller, J., Daly, M.J., 2005. Haploview: analysis and visualization of LD and haplotype maps. Bioinformatics (Oxford, England) 21, 263e265
